# Integrated Proteomics and Metabolomics Analysis in Pregnant Rat Hippocampus After Circadian Rhythm Inversion

**DOI:** 10.3389/fphys.2022.941585

**Published:** 2022-07-22

**Authors:** Jingjing Lin, Xinyue Sun, Xiaofeng Dai, Shaoying Zhang, Xueling Zhang, Qiaosong Wang, Qirong Zheng, Minfang Huang, Yuanyuan He, Rongjin Lin

**Affiliations:** ^1^ School of Nursing Fujian Medical University, Fuzhou City, China; ^2^ The First Affiliated Hospital of Fujian Medical University, Fuzhou City, China; ^3^ Quanzhou Medical College, Quanzhou City, China

**Keywords:** proteomic, metabolomics, hypothalamus, circadian rhythm inversion, pregnant rats

## Abstract

To investigate the changes in proteins, metabolites, and related mechanisms in the hypothalamus of pregnant rats after circadian rhythm inversion during the whole pregnancy cycle. A total of 12 Wistar female rats aged 7 weeks were randomly divided into control (six rats) and experimental (six rats) groups at the beginning of pregnancy. The control group followed a 12-h light and dark cycle (6 a.m. to 6 p.m. light, 6 p.m. to 6 a.m. dark the next day), and the experimental group followed a completely inverted circadian rhythm (6 p.m. to 6 a.m. light the next day, 6 a.m. to 6 p.m. dark). Postpartum data were collected until 7–24 h after delivery, and hypothalamus samples were collected in two groups for quantitative proteomic and metabolism analyses. The differential proteins and metabolites of the two groups were screened by univariate combined with multivariate statistical analyses, and the differential proteins and metabolites enriched pathways were annotated with relevant databases to analyze the potential mechanisms after circadian rhythm inversion. A comparison of postpartum data showed that circadian rhythm inversion can affect the number of offspring and the average weight of offspring in pregnant rats. Compared with the control group, the expression of 20 proteins and 37 metabolites was significantly changed in the experimental group. The integrated analysis between proteins and metabolites found that RGD1562758 and lysophosphatidylcholine acyltransferase 1 (LPCAT1) proteins were closely associated with carbon metabolism (choline, NAD+, L-glutamine, theobromine, D-fructose, and pyruvate) and glycerophospholipid metabolism (choline, NAD+, L-glutamine, phosphatidylcholine, theobromine, D-fructose, pyruvate, and arachidonate). Moreover, the Kyoto Encyclopedia of Genes and Genomes (KEGG) analysis showed that the differential metabolites enriched in adenosine triphosphate (ATP)–binding cassette (ABC) transporters. Our study suggested that circadian rhythm inversion in pregnant rats may affect the numbers, the average weight of offspring, and the expressions of proteins and metabolism in the hypothalamus, which may provide a comprehensive overview of the molecular profile of circadian rhythm inversion in pregnant groups.

## 1 Introduction

Circadian rhythm plays a vital role during pregnancy, and maternal circadian rhythm can affect the fetal formation process (Bates and Herzog 2020). Studies have shown that circadian rhythm inversion can be commonly seen in pregnant groups (Kunzel et al., 2020), and when it happens, the risks of cardiovascular disease, obesity, and other health problems increased in the offspring groups (Seron-Ferre et al., 2012; Varcoe et al., 2018). The hypothalamus in vertebrates is the primary pacemaker of circadian rhythm, which controls the body’s behavior and circadian rhythm, including movement, sleep, body temperature, and endocrine processes (Saper et al., 2005). The hypothalamus, pituitary, and other endocrine organs jointly constitute the hypothalamic-pituitary-other endocrine organs axis, which regulates life activities in the body (Javaheri and Redline 2017). The proteins and metabolites in the hypothalamus may be the beginning of the circadian rhythm inversion to take reaction.

A key switch in the hypothalamus shuts off the activation of the thalamus and the cerebral cortex arousal system during sleep (Chen et al., 2018). Therefore, when the sleeping process is disrupted, this key switch is turbid. Study showed that circadian rhythm inversion can induce the abnormal expression of proteins and metabolisms in the hypothalamus (Phillips et al., 2013). While the effects of circadian rhythm inversion changes on the proteins and metabolites in the hypothalamus after delivery in pregnant rats are not well elucidated, our study used integrated analysis of proteomics and metabolomics to screen out the differential proteins and metabolites in the hypothalamus between the two groups. In addition, we analyzed the possible mechanisms of proteins and metabolites pathways to explore the characteristics of the hypothalamus in pregnant mice with circadian rhythm inversion during pregnancy.

## 2 Materials and Methods

### 2.1 Materials

UA buffer (8 M urea, 150 mM TRIS-HCl pH8.0), ammonia bicarbonate (NH4HCO3, Sigma, A6141), acetonitrile (Merck, 1499230-935), ammonium acetate (Sigma, 73594), methanol (Millipore, 1.06007.4008), and ammonia (Merck, 105426) trypsin (Madison). The ultrasonic cell crusher was purchased from Ningbo Xinzhi Biotechnology Co., LTD. (Ningbo, China). The ultrasonic washing device was purchased from Kunshan Ultrasonic Instrument Co., LTD. (Kunshan, China). A high-speed desktop refrigerated centrifuge was purchased from Beckman Coulter (Brea, United States). A vacuum centrifugal concentrator was purchased from Ebendorf (Saxony, Germany). An electronic analysis balance was purchased from Satrius Tingen, Germany. Thermo Scientific Q-Exactive HF-X was used for the mass spectrometry, Thermo Scientific Easy-NLC1200 was used as the chromatographic system, and a trap column (reverse phase) was used as the chromatographic column, model: 100 μm*20 mm (5 μm, C18), A chromatographic analysis was performed using Thermo Scientific EASY Column (reverse phase), model 75 μm*150 mm, 3 μm, C18.

### 2.2 Model Preparation and Specimen Collection

Several seven-week-old Wistar rats were purchased from Charles River Laboratories (Shanghai, China). The first 24-h adaptive feeding was conducted in an environment where the temperature was maintained at 24 ± 3°C, and natural light was simulated (06:00–18:00). Adequate feed and water were provided. During conception, the pregnant rats were randomly divided into a control group (six rats) or an experimental group (six rats) in the same cage with lamp control equipment. The animals could move freely without the interference of equipment confounding factors. The lamp control device is composed of a LED lamp and a lamp control component, which can control the brightness and time of the light source while providing a simulated natural light source. The physiological characteristics of rats are resting during the day, being active at night, and being sensitive to light and noise. The control group was subjected to a 12-h light and dark cycle light at sunrise and sunset (6 am–6 pm bright and 6 pm–6 am dark the next day), and the intervention group was set with a completely reversed circadian rhythm (6 pm–6 am bright the next day and 6 am–6 pm dark). In both groups, intervention time was from conception to delivery, and water and feed were synchronized. The rats were sacrificed 7–24 h after delivery, and the hypothalamus samples were dissected for metabolisms and proteomics analysis. Our research is strictly in accordance with the Regulations on the Management of Experimental Animals and approved by the Ethics Committee of the First Affiliated Hospital of Fujian Medical University (Number: FJMU-IACUC-2020-0053).

### 2.3 Proteomics: Sample Preparation and Analysis Conditions

#### 2.3.1 Sample Preparation, Protein Enzymatic Hydrolysis, and Peptide Desalting

The samples were remelted at 4°C, and 200 μL SDT lysate was added to each sample, followed by ice bath ultrasound for 2 min and centrifugation at 4°C for 16000 g for 20 min. The supernatant was taken, and protein quantification was performed using the BCA method.

The protein of 300 mg was taken from each sample for FASP enzymatic hydrolysis. The steps were as follows: Add an appropriate amount of 1 M DTT to each sample to reach a final concentration of 100 mM, take a boiling water bath for 5 min, and cool to room temperature. Then add 200 μL UA buffer (8 M urea, 150 mM TRIS-HCl, pH8.0), mix, and centrifuge 12000 g/15 min; add 200 μL UA buffer and centrifuge for 12000 g for 15 min; and discard the filtrate. Add 100 μL IAA (50 mM IAA in UA), shake at 600 rpm for 1 min, avoid light at room temperature for 30 min, and centrifuge at 12000 g for 10 min; add 100μL UA buffer, centrifuge at 12000 g, and repeat twice at 10 min. After that, add 100 μL NH4HCO3 buffer and centrifuge at 14000 g/10 min twice. Add trypsin buffer (6 μg Trypsin in 40 μL NH4HCO3 buffer), shake at 600 rpm for 1 min, 37°C, 16–18 h. A new collection tube was replaced and centrifuged for 12000 g for 10 min. The filtrate was collected, and 0.1%TFA solution was added. After the enzymolysis, the peptide was desalted using a C18 cartridge and freeze-dried in a vacuum. The peptide, after enzymatic hydrolysis, was dried and re-dissolved with 0.1%TFA and quantified by the Thermo Fisher Desalting Spin column for desalting.

#### 2.3.2 TMT Peptide Labeling and Peptide Classification

Peptide was taken from each sample and labeled according to the instructions of the Thermo Fisher TMT Labeling Kit. The labeled peptides in each group were mixed in equal amounts, and the dried peptides were separated using a Pierce™ High pH Reversed-Phase Peptide Fractionation Kit (Thermo Fisher). An LC-MS/MS analysis was performed.

#### 2.3.3 LC-MS/MS

An appropriate amount of peptide was taken from each sample for chromatographic separation using the Easy nLC 1,200 chromatography system (Thermo Fisher). Buffer: Solution A is a 0.1% formic acid aqueous solution, and solution B is a mixture of 0.1% formic acid, acetonitrile, and water (acetonitrile is 95%). The column was balanced with 100% liquid A. The samples were injected into a trap column (100 μm*20 mm, 5 μm, C18, Dr. Maisch GmbH) and then subjected to a gradient separation column (75 μm*150 mm, 3 μm, C18, Dr. Maisch GmbH) at a flow rate of 300 nL/min. The liquid phase separation gradient is as follows: 0–2 min, a linear gradient of liquid B from 2% to 8%. From 2–42 min, the linear gradient of liquid B ranged from 8% to 30%, and 42–49 min, a linear gradient of liquid B from 30% to 45%. At 49–50 min, the linear gradient of liquid B ranged from 45% to 100%. The concentration of liquid B is maintained at 100% for 50–60 min. The peptides were separated and analyzed by the Q-Exactive HF-X mass spectrometer (Thermo Scientific) DDA mass spectrometry. The analysis lasts 60 min, the detection mode is positive ion, the scanning range of the parent ion is 300–1800 m/z, the mass spectrometry resolution is 60,000 @ m/z 200, the AGC target is 3E6, and the maximum IT is 50 ms. The MS2 scan was triggered to collect the 20 highest intensity parent ions after each full scan. MS2 had a resolution of 15,000 @ m/z 200, and the AGC target was 1E5, with a maximum IT of 50 MS. Type of MS2 Activation: HCD, Normalized Collision Energy: 32, Isolation Window: 1.6 m/z.

#### 2.3.4 Untargeted Metabolomics: Sample Preparation and Analysis Conditions

It takes 50 ± 2 magnesium from a hypothalamus sample and adds it to the 2-ml CVD tube and adds 500 μL of CVD 70% methanol and water solution (containing 1 ppm 2-chlorophenyl alanine). The samples were homogenized 4 times, 30 s, 30 HZ each. After homogenization, it was oscillated for 5 min and stood on the ice for 15 min. CVD centrifuge (5427R, Manufactured by Ebender, Germany) was used for 10 min at the CVD C, 12000 r/min, and 400 μL of the supernatant was absorbed into the centrifugal tube. 500 μL ethyl-acetate/methanol (V, 1:3) was added to the precipitate in the centrifuge tube, shaking for 5 min, and standing on ice for 15 min. CVD was centrifugal for 10 min at 4-CVD and 12000 RMP, and 400 μL of the supernatant was taken into the CVD tube. The solution of centrifuge tube 2) and centrifuge tube 3) are combined together and concentrated at room temperature under vacuum. CVD was added with 100 μL 70% methanol and water solution for 3 min ultrasonic, and CVD was centrifugal at CVD C, 12000 r/min for 3 min. CVD 60 μL supernatant was sucked into the CVD inner lining pipe for LC-MS/MS analysis.

Chromatographic conditions: Chromatograph (LC20 Ultra-High Performance Liquid chromatograph, Shimadzu product) was performed on Waters T3 C18 column, I.D. 2.1 × 100 mm, 1.8 μm (C18 WM-13). Mobile phase A was 0.04% acetic acid/water, and mobile phase B was 0.04% acetic acid/acetonitrile. Gradient elution procedure: 0–10 min, linear change 95%–5% A, 5%–95% B; 10–11 min, 5%A, 95%B; 11–11.1 min, linear change 5%–95% A, 95%–5% B; 11.1–14 min, 95%A, 5%B. The CVD column temperature is 40°C. The flow rate was 0.35 ml/min. The injection volume was 2 µL.

Mass spectrometry conditions: The Triple TOF-6600 mass spectrometer (AB Sciex product) adopts electrospray ionization (ESI) positive and negative ion modes for detection. The positive voltage is 5000V, the negative voltage is 4500V, the temperature is 500 °C, the cluster removal voltage is 60V, the collision voltage is 30V, the collision energy dispersion is 15V, and the collision energy is 30 V.

#### 2.3.5 Data Processing

Data integrity and accuracy are necessary to obtain statistically and biologically significant results. Metabolite ion peaks with missing values of more than 50% of the original data were removed without participating in subsequent statistical analysis. The total peak areas of positive and negative ion data were normalized. In addition, Pareto scaling was performed in SIMCA-P software after integrating the positive and negative ion peaks.

The LC-MS/MS RAW files were finally imported into the search engine Sequest HT in Proteome Discoverer software (version 2.4, Thermo Scientific) for database retrieval. The database was Uniprot-Rattus Norvegicus (Rat) [10116]-36180-20210401, from the protein database at https://www.uniprot.org.

### 2.4 Statistical Analysis

Proteomics and metabolomics data are expressed as mean ± SEM. The R package was applied to the volcano map and hierarchical clustering analysis. KEGG net was used to analyze KEGG enrichment in different expressed proteins and metabolites. *p* values less than 0.05 were considered statistically significant.

## 3 Results

### 3.1 Postpartum Situation of Maternal Rats in the Two Groups After Intervention

Pregnant rats in both groups were delivered successfully, and data about pregnancy outcomes were recorded. The results showed that the number of offspring (*p* < 0.05) and the average weight of offspring (*p* < 0.05) decreased in the experiment group, which was treated with circadian rhythm inversion, while the basic weight and postpartum weight were no significant difference between two groups, as shown in Table 1.

**TABLE 1:** Comparison of postpartum conditions in the two groups.

	Control group	Experiment group	Statistic	p-value
Basic weight	214.98 ± 5.47	214.45 ± 5.88	0.18	0.862
Postpartum weight	202.8 ± 23.46	204.48 ± 25.15	0.23	0.821
Number of offspring	10 ± 1.17	6 ± 1.03	6.02	<0.05
Average weight of offspring	5.81 ± 0.12	5.17 ± 0.11	9.49	<0.05

### 3.2 Proteomic Analysis

**FIGURE 1:**
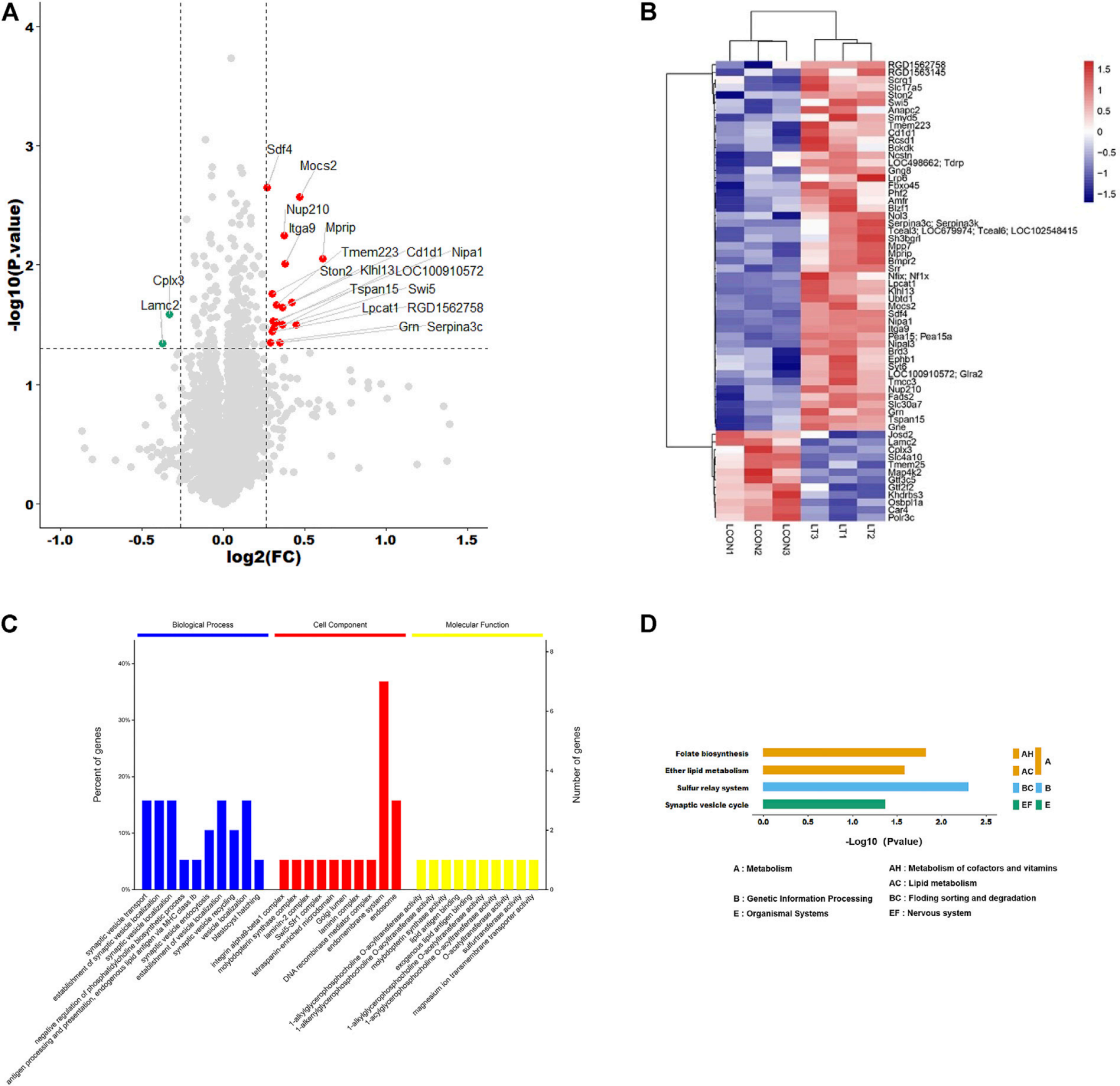
Identification and analysis of differentially expressed proteins. **(A)** Volcano plots presenting the proteins differences between the two groups, and proteins that have a significantly higher level in the experimental group are dotted in red, and those that have a lower level are dotted in green (Fold change >1.2, adjusted *p*-value < 0.05). **(B)** Heat map shows the protein clustering results in two groups (red represents upregulated proteins and blue represents downregulated proteins). **(C)** Significance enrichment analysis of GO annotation in biological process, cell component, and molecular function. **(D)** Based on the KEGG database, the main pathways involved were ether lipid metabolism, folate biosynthesis, sulfur relay system, and synaptic vesicle cycle in the network.

Proteome Discoverer software was used for qualitative analysis of TMT marker proteomics data, and a total of 6648 proteins were identified. Compared with the control group, the expression levels of 20 proteins were significantly changed. The volcano diagram and heatmap showed that 18 proteins were significantly upregulated (FC > 1.2, *p* < 0.05) and two were significantly downregulated (FC > 1.2, *p* < 0.05) (Figures 1A,B). The significance enrichment analysis of GO annotation was used to evaluate the significance level of protein enrichment in a certain GO term using Fisher’s precise test, which demonstrated that these differential proteins were mainly involved in synaptic vesicle transport in biological processes, endomembrane system in cell components, and 1-acylglycerophosphocholine O-acyltransferase activity in the intervention group vs. control group (Figure 1C). Based on the KEGG database, the main pathways involved were Folate biosynthesis, Ether lipid metabolism, Sulfur relay system, and Synaptic vesicle cycle in the network (Figure 1D).

### 3.3 Metabolomics Analysis

**FIGURE 2:**
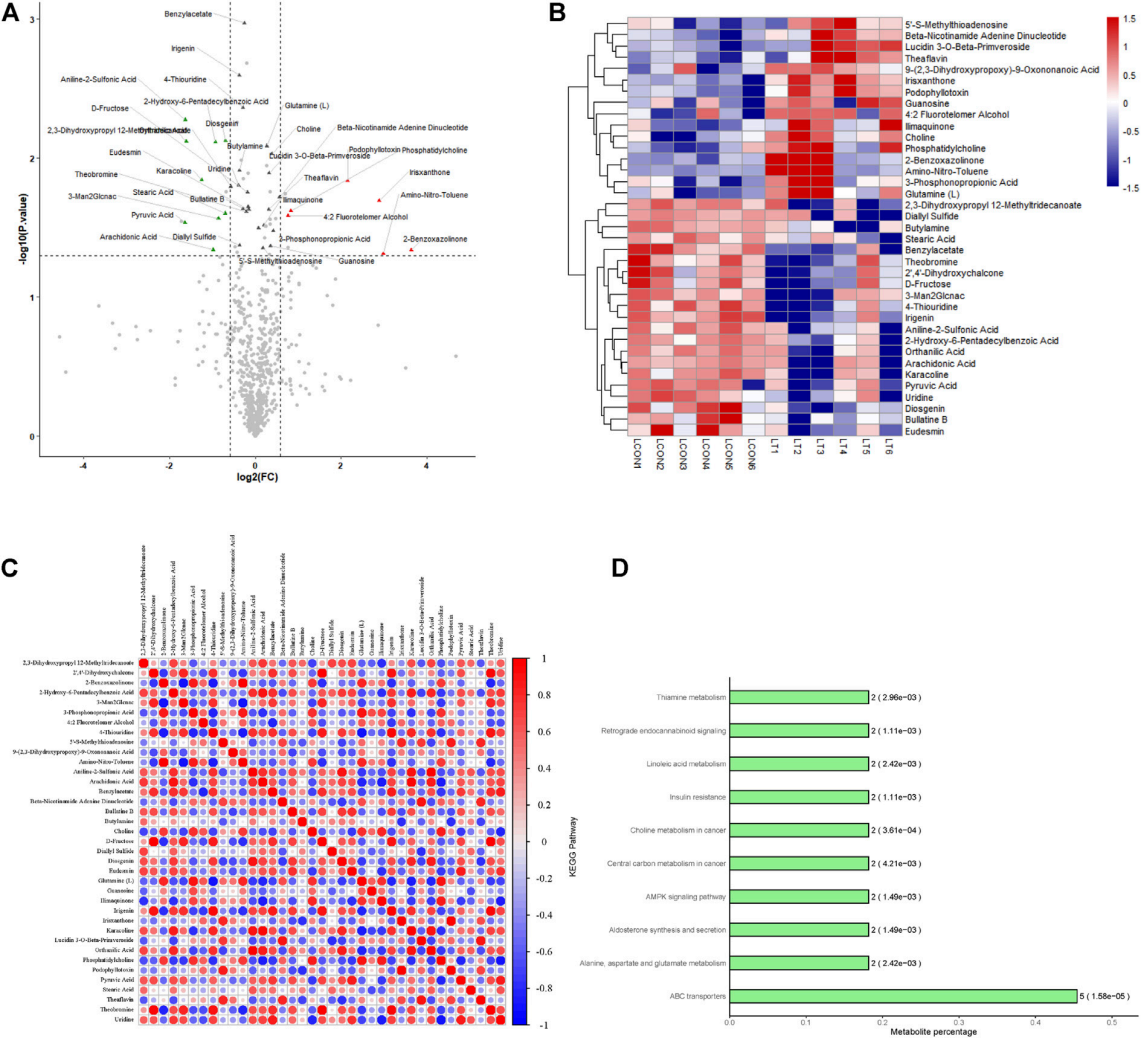
Identification and analysis of differentially expressed metabolites. **(A)** Volcano plots presenting the metabolites differences between two groups; red plots represent significantly upregulated metabolites and green represents downregulated metabolites. **(B)** Heat map shows the metabolites clustering results in two groups (red represents upregulated proteins and blue represents downregulated proteins). **(C)** Heat map of the correlation coefficient matrix demonstrated the correlations between the significant metabolites. **(D)** Metabolites obtained in the comparison group were enriched in the KEGG metabolic pathway.

A total of 696 metabolites were identified in this experiment, and the expressions of 10 metabolites were significantly changed by OPLS-DA with VIP >1 and *p* < 0.05 as the threshold (Table 2). The volcano diagram and heatmap were used to assess the expression levels of the metabolites found in two groups, in which 5 metabolites were significantly upregulated (FC > 1.5, *p* < 0.05), and five metabolites were significantly downregulated (FC > 1.5, *p* < 0.05) (Figures 2A,B). The heatmap of the correlation coefficient matrix demonstrated the correlations between the significant metabolites (Figure 2C). The metabolites obtained in the comparison group were enriched in the KEGG metabolic pathway (Figure 2D).

**TABLE 2:** Significant metabolites and related pathways.

m/z	RT (min)	VIP	Metabolite name	Formula	FC	p-value	KEGG pathways ID
325.23495	2.876	2.35683	2,3-Dihydroxypropyl-12-methyltridecanoate	C17H34O4	0.671782926	0.015971806	
239.07831	13.627	5.53845	2′,4′-Dihydroxychalcone	C15H12O3	0.890850575	0.022284846	
136.03911	4.363	3.88633	2-Benzoxazolinone	C7H5NO2	12.46643677	0.046025831	
347.2572	3.735	3.34726	2-Hydroxy-6-pentadecylbenzoic acid	C22H36O3	0.525867847	0.007686917	
546.2019	14.035	1.3337	3-Man2Glcnac	C20H35NO16	0.551330883	0.027091806	
152.9962	9.152	2.23056	3-Phosphonopropionic acid	C3H7O5P	1.33697394	0.033196058	
263.01355	13.095	5.54754	4:2 Fluorotelomer alcohol	C6H5F9O	1.699333892	0.026009249	
261.0582	13.612	1.70449	4-Thiouridine	C9H12N2O5S	0.821459239	0.004308547	
298.09698	4.848	4.88472	5′-S-Methylthioadenosine	C11H15N5O3S	1.13033054	0.044174232	
263.14502	12.587	2.57388	9-(2,3-Dihydroxypropoxy)-9-oxononanoic acid	C12H22O6	1.053907846	0.031780433	
153.0658	4.366	5.70612	Amino-nitro-toluene	C7H8N2O2	7.954082137	0.048454571	
172.00697	5.818	1.42617	Aniline-2-sulfonic acid	C6H7N	0.321732416	0.005285527	
305.24466	1.803	3.33309	Arachidonic acid	C20H32O2	0.507101399	0.045601456	map05140,04666,04750,04270
149.06129	3.701	1.33265	Benzylacetate	C9H10O2	0.837589461	0.001062728	
664.11188	14.02	1.4117	Beta-Nicotinamide adenine dinucleotide	C21H27N7O14P2	1.483947385	0.019033915	map01100,00983,00760,00730
460.26794	2.884	1.22504	Bullatine B	C24H39NO6	0.821540349	0.023155541	
74.09618	2.909	2.07637	Butylamine	C4H11N	0.777459099	0.01221642	
104.10668	9.763	7.35948	Choline	C5H14NO	1.308167562	0.009307603	map01100,00564,02010,04976,00260
179.05692	13.627	6.98592	D-Fructose	C6H12O6	0.880392392	0.017544554	map01100,04973,02010,00051,00052
136.93945	12.107	9.54638	Diallyl sulfide	C6H10S	0.776001868	0.042349612	
437.29877	4.366	2.21911	Diosgenin	C27H42O3	0.619112294	0.007484634	
387.17889	1.324	5.09526	Eudesmin	C22H26O6	0.902264921	0.023488135	
169.05676	13.687	2.72515	Glutamine (L)	C5H10N2O3	1.202091666	0.008152464	map01100,00630,00220,00230,004964
284.09875	9.502	3.22387	Guanosine	C10H13N5O5	1.283647062	0.042913451	map01100,02010,00230
433.00113	13.089	5.27059	Ilimaquinone	C22H30O4	1.150297627	0.030278805	
359.12033	13.624	1.89156	Irigenin	C18H16O8	0.771759379	0.002532267	
435.1011	0.686	5.79341	Irisxanthone	C20H20O11	7.42498619	0.02020363	
378.26279	1.765	2.16532	Karacoline	C22H35NO4	0.419813304	0.014298142	
563.12439	13.98	1.98545	Lucidin-3-O-beta-primeveroside	C26H28O14	1.253246375	0.012793306	
174.02257	5.815	1.41516	Orthanilic acid	C6H7NO3S	0.327011971	0.0075562	
796.5451	6.164	2.24383	Phosphatidylcholine	C43H76NO8P	1.780459777	0.023969459	map01100,00564,00590,00591,00592
437.11407	0.691	1.93554	Podophyllotoxin	C22H22O8	4.451590913	0.014517779	
87.00893	6.105	2.33681	Pyruvic acid	C3H4O3	0.321061012	0.028938203	map00430,00270,00040,01200
283.26309	1.859	3.20129	Stearic acid	C18H36O2	0.615216583	0.024925296	map00061,01040
565.13745	13.977	1.51817	Theaflavin	C29H24O12	1.244116434	0.023455134	
198.09807	13.619	1.59168	Theobromine	C7H8N4O2	0.863001625	0.024294497	map01100,00232
243.06143	6.213	13.8741	Uridine	C9H12N2O6	0.765153296	0.015757169	map00240,01100,02010

### 3.4 Combination of Proteomics and Metabolomics

**FIGURE 3:**
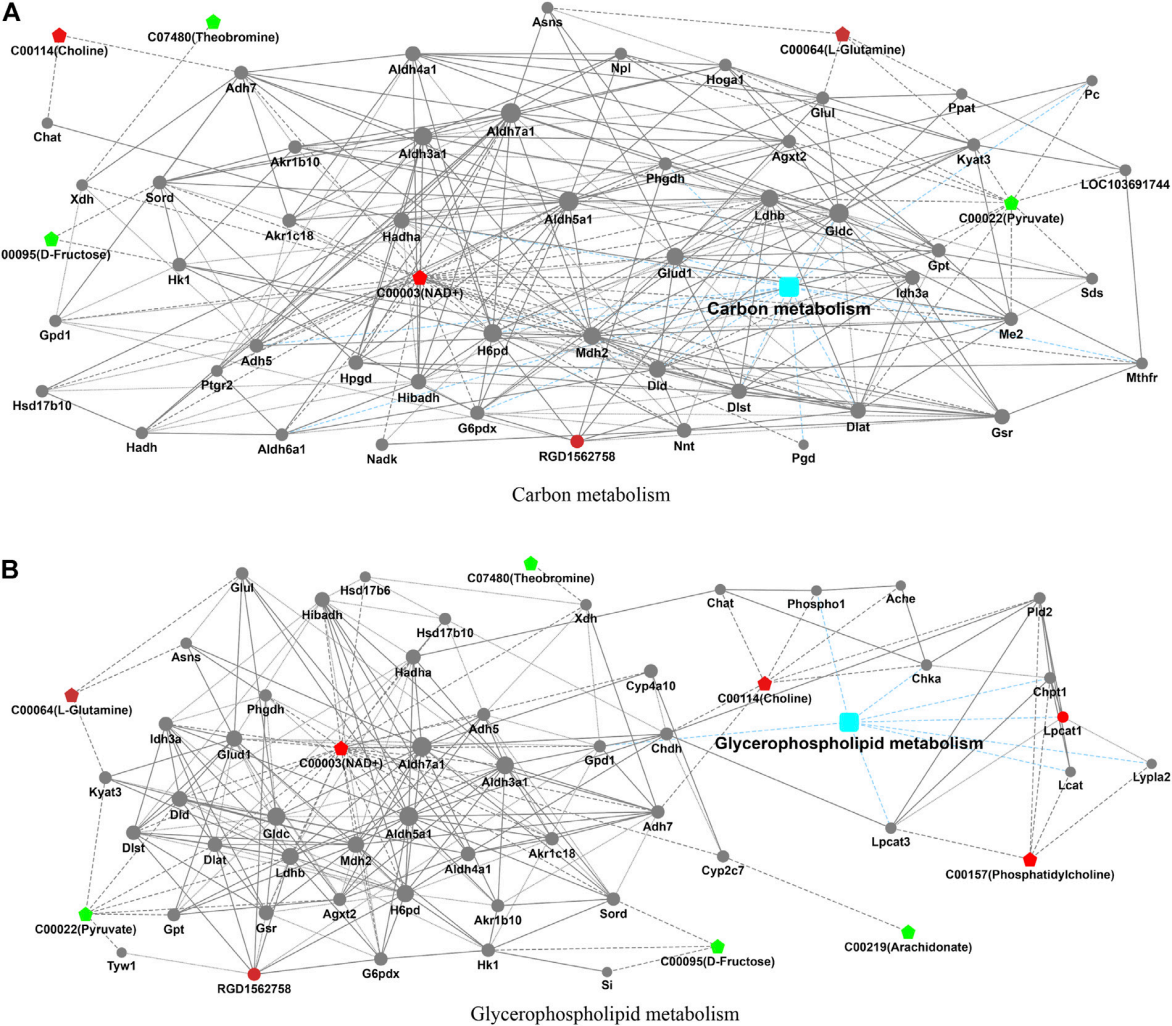
Integrated analysis of proteomics and metabolomics. The integrated pathways analysis results of proteomics and metabolomics showed that carbon metabolism pathway **(A)** and glycerophospholipid metabolism pathway **(B)** are the relevant pathway of integration analysis. (boxes represent proteins, circles represent metabolites, red represents upregulation, and blue represents downregulation).

In the process of metabolism, the expression trend of metabolites is linked to the expression level of metabolic enzymes. Metabolism data were analyzed in conjunction with proteomics data. The results found that carbon metabolism and glycerophospholipid metabolism pathways that included significantly altered proteins (RGD1562758 and Lpcat1) and metabolites (choline, NAD+, L-glutamine, theobromine, D-fructose, pyruvate, phosphatidylcholine, and arachidonate) were found (Table 3; Figure 3).

**TABLE 3:** Significantly altered pathways with differentially expressed metabolites and proteins.

No	Pathway name	Metabolites	Proteins
1	Carbon metabolism	Choline and NAD+	RGD1562758 and LPCAT1
		L-Glutamine and theobromine	
		D-Fructose and pyruvate	
2	Glycerophospholipid metabolism	L-Glutamine and NAD+	RGD1562758
		Choline and phosphatidylcholine	
		Theobromine and pyruvate	
		D-Fructose and arachidonate	

## 4 Discussion

Regular sleep is vital to human physiological and mental health, especially for pregnant women (Tsai et al., 2021). The study showed that about 45.7% of pregnant women were prone to circadian rhythm inversion (Sedov et al., 2018). The circadian rhythm inversion may lead to biochemical and neurophysiological dysfunction, which may increase uterine contraction force to increase the risk of premature birth (Palmer et al., 2013; Fernandez et al., 2016). It results in a profound influence on maternal and fetal health and even postpartum maternal and offspring physical and mental health. Consistent with these studies, our research found that circadian rhythm inversion can affect the number of offspring and the average weight of offspring. In addition, the integrated proteomics and metabolomics analysis found that circadian rhythm inversion changes the expression of LPCAT1, RGD1562758 proteins, and carbon metabolism and glycerophospholipid metabolism via the ABC transport pathway. Therefore, more attention should focus on the effects of circadian rhythm inversion during pregnancy.

The transcriptional molecular circadian clocks are present in organs, tissues, and even in individual cells, where they exert circadian control over cellular metabolism, including carbon metabolism and glycerophospholipid metabolism (Klemz et al., 2017) (Sosa Alderete et al., 2020). Glycerophospholipid metabolism is the main lipid constituent of cell membranes, directly affecting cell physiological functions and forming the basis for creating dynamic sub-compartments within membranes (Zong et al., 2018). Changes in the concentration of glycerophospholipids indicate transformations in cell membrane composition and permeability. The synthesis and degradation of glycerophospholipids are the most highly regulated metabolisms across the 24 h cycle in terms of total lipid content and enzyme expression and activity in individual cells (Guido et al., 2022). It has been confirmed that the functions of glycerophospholipid metabolism in the nucleus is modulated in response to diverse external cues that impact nuclear functions (DNA methylation, histone modifications, chromatin structure, etc.) with high significance in fetus developments (Lewis and Aitken 2005; Harayama and Riezman 2018). The reduction of peroxidase glycerophospholipid metabolism to replace the oxidized sn-2 fatty acyl group through LPCATs can provide a complete system for repairing peroxidase cell membranes (Fisher 2018). LPCATs are essential enzymes that regulate phospholipid metabolism and mediate the synthesis and remodeling of phospholipids in cells through deacylation (Wang and Tontonoz 2019; Lin et al., 2021). The primary function of LPCAT1 is to mediate the deacetylation of lysophosphatidylcholine in the saturate phosphatidylcholine. LPCAT1 can regulate lipid droplet formation. Lipid droplets are intracellular organelles that store neutral lipids, provide energy for membrane synthesis, and signal lipid production (Bai et al., 2021). Lipid droplets play an indispensable role in regulating biological processes such as lipid storage, fatty acid transport, and transcription factor activation (Filali-Mouncef et al., 2022). Membrane remodeling mediated by abnormal phospholipid metabolism drives the continuous activation of the cell growth factor signaling pathway. A study conducted by Chassen et al. (Chassen et al., 2018) further confirmed that the turbulence of lipid droplets in pregnant can restrict fetus growth in placental. It provides a new perspective for understanding the mechanism of circadian rhythm inversion.

Carbon metabolism, medicated by the folate cofactor, support multiple physiological processes (Ducker and Rabinowitz 2017). In pregnancy procession, it causes a disposition to birth defects known as neural tube defects, which involve failure of neural tube closure early in pregnancy, and the outcomes range in severity from anencephaly, causing fetal loss (Copp et al., 2015). RGD1562758 protein is involved in both carbon metabolism and the glycerophospholipid metabolism pathway. RGD is a tripeptide sequence containing arginine, glycine, and aspartic acid (Zhang et al., 2020). It is the recognition site of the interaction between integrins and ligand proteins, mediates the adhesion between cells and extracellular matrix and between cells, and has a signal transduction function, thus mediating many essential life activities (Barczyk et al., 2010). Dumont et al. (2019) demonstrated that the turbulence expression of RGD1562758 can affect carbon metabolism, and eventually lead to the disorder of energy metabolism which is harmful to the body. Metabonomic analysis showed that carbon and glycerophospholipid are important metabolic differential substance and closely related to the expression of RGD156278 (Stroumpoulis et al., 2007; Rice et al., 2011). The study demonstrated that changes in glycerophospholipid metabolism are closely related to the lack of sleep caused by the reversal of circadian rhythm in pregnant rats (Wiesenfeld et al., 2006). Glycerophospholipid are critical structural and functional components of cell membranes. Various amino alcohols can participate in synthesizing glycerophospholipid in the body, and choline can also be transformed into other substances through demethylation. This change in insulin sensitivity may be closely related to compounds involved in changes in glycerophospholipid metabolism (Depner et al., 2020).

The KEGG metabolic pathway results revealed that the differential metabolites were enriched in the ABC transporter pathway. The ABC transporter is a highly conserved cell transmembrane transporter with a high expression level in monocytes and macrophages, partially regulated by sterol flow (Schmitz et al., 2000). A and G transporters in the ABC family tend to be downregulated in the absence of sleep (Aho et al., 2016). Depner CM et al. (2020) pointed out that based on blood metabolomics lipid homeostasis, high-density lipoprotein remodeling, and plasma lipoprotein remodeling, biochemical pathways were activated after human sleep deficit. In this study, the significant changes in glutamine, uridine, and guanine nucleoside metabolites also clearly pointed out the ABC transporter signaling pathway changes, which is consistent with the results of this study.

## 5 Conclusion

In summary, our study found that inversion of circadian rhythm in pregnant rats can promote the expression of RGD1562758 and LPCAT1 proteins. Furthermore, circadian rhythm inversion is closely related to the disorder of carbon metabolism (choline, NAD+, L-glutamine, theobromine, D-fructose, and pyruvate) and glycerophospholipid metabolism (L-glutamine, NAD+, choline, phosphatidylcholine, theobromine, pyruvate, D-fructose, and arachidonate) *via* ABC transporter pathway in pregnant rats. The results of our study suggest that the disorder proteins and metabolisms may be key biomarkers and potential therapeutic target in future studies on circadian rhythm inversion in pregnant women.

## Data Availability

The data presented in the study are deposited in the iProX repository with the accession number IPX0004631000 and in MetaboLights with the accession number MTBLS5026.
